# Association of traumatic events in childhood, impulsivity and decision-making with previous suicide attempt and/or current suicidal ideation in adult patients with major depressive disorder

**DOI:** 10.1192/j.eurpsy.2021.472

**Published:** 2021-08-13

**Authors:** A. Roman, J. Rodríguez-Revuelta, F. Dal Santo, L. García-Alvarez, L. De La Fuente-Tomás, C. Martínez-Cao, L. Jiménez-Treviño, L. González-Blanco, M.P. García-Portilla, J. Bobes, P.A. Saiz

**Affiliations:** 1 Departament Of Psychiatry And Clinical Psychology, Clínica Universidad de Navarra, Pamplona, Spain; 2 Neuroscience And Sense Organs, ISPA HEALTH RESEARCH INSTITUTE OF THE PRINCIPALITY OF ASTURIAS, Oviedo, Spain; 3 Psychiatry, SESPA Mental Health Services of Principado de Asturias, OVIEDO, Spain; 4 Department Of Psychiatry, University of Oviedo, Oviedo, Spain; 5 Psychiatry, Hospital Universitario Central de Asturias, Oviedo, Spain; 6 Psychiatry, CIBERSAM, Madrid, Spain

**Keywords:** childhood trauma, impulsiveness, Decision-making, Suicide

## Abstract

**Introduction:**

Suicidal behavior has a great impact on world public health. The literature describes the possible existence of an association between neurobiological, clinical and cognitive factors in suicidal behavior.

**Objectives:**

To determine the possible relationship between clinical variables (history of abuse/maltreatment in childhood), psychopathology (impulsivity traits) and cognitive (decision-making) with a history of suicide attempt and/or current suicidal idea in patients with major depressive disorder.

**Methods:**

Cross-sectional study in a sample of adult patients with major depressive disorder in which two types of comparisons are made. In the first case, two groups were compared based on the presence or absence of history of suicide attempt. In the second case, two groups were compared based on the presence or absence of suicidal ideation in the same sample of patients. Finally, sociodemographic, clinical and cognitive variables were evaluated in that population sample.

**Figure:**
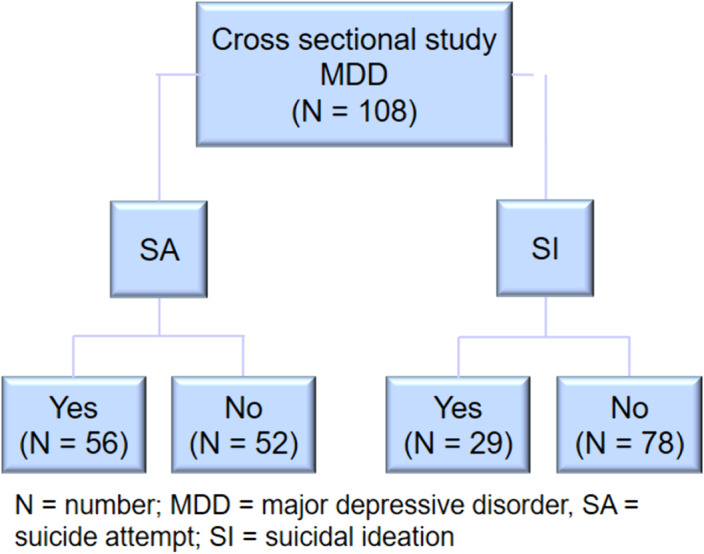

**Results:**

When the joint influence of sociodemographic, clinical and cognitive characteristics are present, it can be said that being single/divorced/separated, a history of sexual abuse in childhood and an alteration in decision-making, specifically a lower number of choices of deck D in the IGT test, are associated with a higher probability of a personal history of suicide attempt. While a higher score on the Barrat impulsivity scale is associated with a greater probability of presenting current suicidal ideation once the influence of sociodemographic, clinical and cognitive variables has been taken into account.

**Figure:**
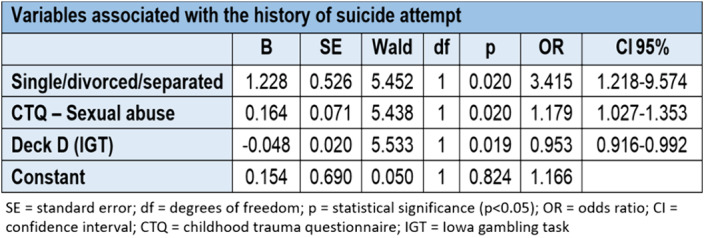

**Figure:**
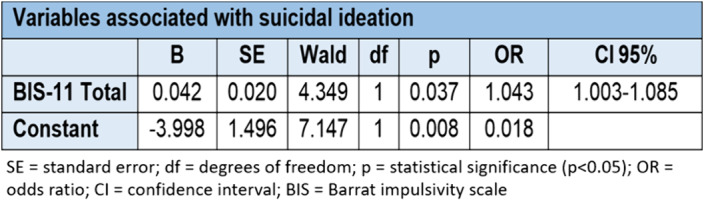

**Conclusions:**

Different sociodemographic, clinical and cognitive factors are associated with the presence of a history of suicide attempt and/or current suicidal ideation.

**Disclosure:**

No significant relationships.

